# Incidence of COVID-19-Associated Venous Thromboembolism Among Hospitalized Patients in McAllen, Texas, USA, in Late 2021

**DOI:** 10.7759/cureus.23270

**Published:** 2022-03-17

**Authors:** Parvaneh Bashardoust, Benjamin J Fano

**Affiliations:** 1 Internal Medicine, Oceania University of Medicine, Apia, ASM; 2 Internal Medicine, South Texas Health System, McAllen Medical Center, McAllen, USA

**Keywords:** hospitalized patients in mcallen, mcallen-texas, covid-associated vte, texas covid incidence, covid-19 complications, covid-19, venous thromboembolsim

## Abstract

This study investigated the incidence of venous thromboembolism (VTE) in hospitalized COVID-19 patients in a community hospital in McAllen, Texas, USA. Such incidence was reported to be as high as 31% in early 2020, and in the range of 3.1%-13.6% in mid-2020, with no later studies addressing this issue. We identified a total of 47 COVID-19 hospitalized patients during August 2021, among whom four (8.5%) had a documented VTE. They were all on prophylactic anticoagulation from the time of admission, and none of them had disseminated intravascular coagulation (DIC) or a prior history of VTE. The incidence was equal between ICU and non-ICU patients. Pre-existing hypertension and cardiovascular diseases, but not high body mass index (BMI) or diabetes mellitus, appeared to be among risk factors for VTE in these patients. All four VTE patients were of Hispanic ethnicity, while only half of all 47 patients were Hispanic. The study concluded that in late 2021 the rate of VTE remained to be higher in COVID-19 than non-COVID-19 patients in hospitals despite routine and early implementation of prophylactic anticoagulation in this patient population.

## Introduction

It is well known that hospitalized patients are at increased risk of venous thromboembolism (VTE) for a variety of reasons [1]. It is estimated that in non-surgical patients hospitalized for an acute medical illness, the incidence of VTE is more than 100 times greater than the general public [2]. Among others, older aged people and males appear to have important risk factors for hospital-associated VTE [2]. Moreover, patients admitted to the intensive care unit (ICU) are at higher risk of VTE than non-IUC patients [3]. In three prospective studies between 1980 and 2000, researchers found a VTE incidence of 30% when prophylactic anticoagulation was not routinely administered to hospitalized patients to medical ICU patients [4-6]. Later studies demonstrated that the rate of VTE in hospitalized patients can be reduced to 2%-4% with routine use of prophylactic anticoagulation [7].

A state of hypercoagulability with an increased incidence of VTE has been reported in hospitalized patients with coronavirus disease 2019 (COVID-19). The pathogenesis of severe hypercoagulability in COVID-19 patients is not fully understood. Endothelial injury, increased fibrinogen, and factor VIII levels, as well as von Willebrand factor activity, have been implicated in COVID-19 associated coagulopathy (CAC). In addition, circulating thrombotic microparticles, increased polyclonal gamma globulins with a resultant hyperviscosity, and abnormal platelet activation is involved in this process [8-14]. In contrast to DIC, the prothrombin time (PT) and partial thromboplastin time (PTT) are normal or mildly elevated, and platelet count is normal in CAC.

A meta-analysis that included 42 studies enrolling 8271 inpatient cases reported an overall rate of VTE of 21% in COVID-19 patients, with 5% among non-ICU patients and up to 31% in the ICU setting [15]. Another meta-analysis which included 36 studies, reported a VTE incidence of 28% in COVID-19 patients in ICU when diagnostic studies were done for patients with clinical findings suspicious for VTE [16]. Studies have also shown a predominance of male gender among COVID-19 patients with VTE and a high prevalence of obesity, as well as other chronic medical comorbidities, including hypertension, other cardiovascular diseases, and diabetes [17,18].

The above studies were mostly conducted during the first three months of 2020, which was early in the course of the COVID-19 pandemic. Interestingly, studies conducted closer to mid-2020 reported a significantly lower incidence of VTE, as low as 3.1%-13.6% [19-21]. The reduction of this incidence from 21%-28% in early 2020 to 3.1%-13.6% in mid-2020 may have been due to the more widespread and routine use of prophylactic anticoagulation in this patient population or advances in the management of the disease. Data regarding the incidence of VTE in hospitalized COVID-19 patients during and after the second half of 2020 are lacking. It is unclear, despite current knowledge of hypercoagulability in COVID-19 patients and more advanced management strategies for this disease, whether VTE is still a significant threat to the hospitalized COVID-19 patients or the risk is now similar to other patients.

In this study, we investigated the incidence of VTE and its association with the above-mentioned comorbidities in COVID-19 patients hospitalized in McAllen Medical Center Hospital, McAllen, Texas, USA, during August 2021.

## Materials and methods

The study was approved by the Institutional Review Board (IRB) of the Oceania University of Medicine. In this retrospective study, patients with a diagnosis of COVID-19 who were hospitalized during a high variant episode in the month of August 2021 in McAllen Medical Center Hospital, McAllen, Texas, USA, were identified. Through the secure electronic medical records system, patient charts were reviewed retrospectively. The following information was recorded for each patient: gender, age, ethnicity, weight, height, BMI, history of hypertension, history of cardiovascular disease, a history of diabetes, range of PT/international normalized ratio (INR), PTT, platelet count, d-dimer, hospital unit (ICU vs non-ICU), and tests for VTE, including doppler-ultrasound of extremities, chest CT scan, and V/Q scan. The inclusion criteria were age 18 years and above, a confirmed diagnosis of COVID-19 on admission or during hospitalization, and a diagnosis of deep venous thrombosis (DVT) and/or pulmonary embolism (PE) on admission or during hospitalization confirmed by an imaging study. The exclusion criteria were a history of VTE prior to admission and a diagnosis of superficial venous thrombosis without DVT and/or PE. Following completion of the chart review process, the cases were numbered and de-identified. Patients with at least one episode of VTE were identified. The percentages were calculated using the paper and pencil method with a basic calculator. The formula used was dividing the value of interest by the total value, and multiplying the results by 100. The medians were calculated using paper and pencil with a basic calculator utilizing the standard formula. 

## Results

The hospital admitted only adult patients; therefore, the studied patients were 18 years or older. A total of 47 patients were included in the study, 24 males and 23 females (Table 1). The age range was 19-89 years, with a median of 52. Thirty-one patients had at least one imaging study, including doppler-ultrasound of extremities and/or V/Q scan and/or CT scan of the chest performed during their course of hospitalization. Four patients (8.5%) had a documented VTE, including two deep venous thrombosis (DVT) and two pulmonary embolisms (PE). These patients were in the age range of 46-65 years with a median of 55, while the median age for non-VTE patients was 51. All four patients with VTE were Hispanic, while half of all patients were non-Hispanic. Among 37 patients on the regular floor and 10 in ICU, three (8%) and one (10%) had a VTE, respectively. The median BMI for the non-VTE patients and VTE patients was 38.0 and 31.8, respectively. Twenty-two patients (50%) without VTE and three (75%) with VTE had a history of hypertension. Ten patients (23%) without VTE and two (50%) with VTE had a history of cardiovascular disease. Twenty patients (47%) without VTE and 2 (50%) with VTE had a history of diabetes. The median highest INR/PTT during hospitalization in VTE and non-VTE patients were 1.4/33.2 and 1.02/26.1, respectively. The median lowest platelet count was 165,000 and 253,000 for the VTE and non-VTE patients, respectively. The median d-dimer was 6713 ng/ml in patients with VTE and 1791 ng/ml in those without VTE.

**Table 1:** Patient characteristics BMI: body mass index, HTN: hypertension, CVD: cardiovascular disease, DM: diabetes mellitus, INR: international normalized ratio, PT: prothrombin time, PTT: partial thromboplastin time * Highest number for individual patients during their hospitalization ** Lowest number for individual patients during their hospitalization

	Patients without VTE (total of 43)	Patients with VTE (total of 4)	All patients (total of 47)
Age	51 (19-89)	55 (46-65)	52 (19-89)
Female, Male	21, 22	2, 2	23, 24
Hispanic	20	4	24
Non-Hispanic	23	-	23
ICU	9	1	10
Non-ICU floor	34	3	37
Height (cm)	165 (145-190)	170.5 (157.5-175.5)	165.5 (145-190)
Weight (kg)	89.8 (44-170)	93 (73-116) kg	90 (44-170)
BMI	33.4 (19.57-73.19)	31.8 (27.93-38.25)	33.2 (19.57-73.19)
History of HTN	22	3	25
History of CVD	10	2	12
History of DM	20	2	22
Highest PT/INR*	1.02 (0.93-1.66)	1.4 (1.02-1.83)	1.05 (0.93-1.83)
Highest PTT*	26.1 (20.1-44.9)	33.2 (27-49)	27 (20.1-49)
Lowest platelet**	253 (67-442)	165 (119-201)	245 (67-442)
Highest d-dimer*	1791 (418 - >10,000)	6713 (510 - >10,000)	2296 (418 - >10,000)

## Discussion

Multiple large studies reported an unusually high rate of approximately 30% of VTE in hospitalized COVID-19 patients during the first few months of 2020, and a state of hypercoagulability has been described with this infection. Subsequently, more stringent prophylactic anticoagulation measures were adopted by hospitals. Shortly later, around March-April 2020, reports from New York, Boston, and New Orleans indicated a significant decline in the rate of VTE in this patient population, down to 3.1%-13.6%, possibly due to more widespread prophylactic anticoagulation strategies and improved management of the COVID-19 infection [19-21]. All studies reported a significantly higher VTE rate in ICU patients than those on regular floors. This can be explained by the fact that ICU patients are less mobile and generally carry more complications.

Reports regarding the rate of VTE in hospitalized COVID-19 patients during the second half of 2020 and the year 2021 are lacking. We looked at this issue more than a year after previous studies to address the state of VTE incidence in this patient population in late 2021. In this study, patients admitted to a community hospital in the city of McAllen, Texas, USA (population of 143,000) in August 2021 during a COVID-19 out-break infection were retrospectively reviewed. Among 47 patients, four (8.5%) had a documented VTE. This is in accordance with the second set of reports in 2020. All these patients had been placed on prophylactic anticoagulation upon admission to the hospital, and none had a prior history of VTE. Such finding emphasizes that aggressive prophylactic anticoagulation plays an important role in reducing the rate of VTE in hospitalized COVID-19 patients. It also suggests that despite advancements in the management of this infection and the clear knowledge of the COVID-19-associated hypercoagulability, the incidence of VTE has not further declined in these patients over more than a year and that despite prophylactic measures, VTE remains a serious threat to this patient population. This finding also reiterates that a constant suspicion or perhaps a vigilant screening for VTE may be required while caring for these patients.

Previous studies had reported a male predominance in COVID-19 patients with VTE; however, our study did not observe a gender preference. The rate of VTE was considerably higher in ICU patients on previous reports, but in our study, there was no significant difference between ICU and non-ICU settings (1/10 [10%] in ICU and 3/37 [8%] in non-ICU). To our knowledge, prior studies did not address a possible role for ethnicity in this phenomenon. In our study, all VTE patients were Hispanic, while the number of non-Hispanic patients on the study was slightly higher than Hispanics (23 vs. 20, respectively). Although there are assumptions of inequality of care between ethnicities in the outpatient setting, our patients were all treated the same while in the hospital regardless of their ethnic background. Such findings may suggest a propensity for certain ethnicities to be at higher risk of VTE during COVID-19 infection. We examined the role of BMI since a high BMI could be a risk factor. The median BMI of VTE patients was indeed slightly lower than that of non-VTE patients (31.8 vs. 33.4, respectively). The BMI appeared to be fairly distributed in non-VTE patients in the range of 22-44, but there were five outliers with BMIs of 19.57, 20.8, 48.95, 54.8, and 73.19. When these five cases were censored, the median BMI of non-VTE patients was 32.1, which still came similar to the VTE patients.

Previous studies reported pre-existing hypertension, cardiovascular diseases, and diabetes to be associated with a higher incidence of VTE in this patient population. In our patients, pre-existing diabetes was equally distributed among VTE and non-VTE patients, but there was a higher rate of hypertension and cardiovascular diseases among those with VTE (3/4 [75%] in VTE patients, and 22/43 [50%] in non-VTE patients for hypertension; 2/4 [50%] in VTE patients, and 10/43 [23%] in non-VTE patients for cardiovascular disease). However, the small number of our VTE patients makes a firm conclusion difficult.

In DIC, the PT/INR and PTT are invariably elevated, and the platelet count commonly decreased, while in COVID-19-associated coagulopathy, these tests are usually around normal values. We looked at the highest PT/INR and PTT and the lowest platelet count recorded during hospitalization to distinguish possible DIC from COVID-19-associated coagulopathy. In the VTE patients, we limited this to the period before the diagnosis of VTE since full-dose heparinization after VTE diagnosis could have affected the coagulation tests. The median PT/INR, PTT, and lowest platelet count were within the normal range for all patients regardless of their VTE status. One VTE patient had mildly elevated coagulation tests at baseline (INR 1.83, PTT 49) and a platelet count of 148, still not abnormal enough to be suspicious for a clinically significant DIC. In addition, none of the VTE patients had moderate or severe thrombocytopenia to suggest possible heparin-induced thrombocytopenia.

Finally, we reviewed the recorded d-dimer levels in all studied patients. It is known that the d-dimer level is commonly elevated in COVID-19 patients [22]; also, an elevated d-dimer is a frequent finding in patients with an acute VTE. Consistent with such knowledge, the median d-dimer in VTE versus non-VTE patients was significantly higher at 6713 ng/ml versus 1791 ng/ml, respectively.

The findings of this study, although at a smaller scale, are comparable with previous reports near mid-2020 when the incidence of VTE in hospitalized COVID-19 patients was at 3.1%-13.6%. This is, to our knowledge, the first study examining such incidence after the initial months of the COVID-19 pandemic. The significance of this study is to highlight the fact that despite advances made in the management of patients hospitalized with COVID-19 infection, and the preemptive anticoagulation measures, VTE remains a real threat in this setting. Practitioners need to remain on high alert for signs and symptoms of VTE in this patient population and perhaps consider more aggressive prophylaxis strategies than what is routinely implemented. Additionally, the study was conducted in a small-town local hospital, reflecting typical daily practice and related challenges in non-academic less-resourceful facilities. Moreover, an interesting finding in this study was that all patients with VTE were of Hispanic ethnicity even though they had received the same prophylactic measures as others.

There were limitations to this study. The study data were related to a community hospital in a city in Southern Texas, and the sample size was not as large as previous reports. The study, however, reflected a real-life situation, which was a common daily practice in a local hospital during an overwhelming COVID-19 surge. The number of VTE patients in this study was small, which makes it difficult to make clear conclusions regarding the association of VTE with the above-mentioned risk factors. Nevertheless, the study addressed a common and important complication of COVID-19 infection, and it appears that despite advancements in the management of this disease, the rate of VTE has not declined longer than a year after previous reports. This study also highlights the importance of awareness regarding the risk of VTE in hospitalized COVID-19 patients, the need for prophylactic anticoagulation, early ambulation, and emphasis on the outpatient management of this disease. Future prospective studies may consider the role of more aggressive prophylactic anticoagulation strategies than what is currently implemented to further decrease the risk of VTE in this patient population.

## Conclusions

This study reflects the common daily medical practice in a local community hospital. In this setting, despite routine and aggressive prophylactic anticoagulation measures, the rate of VTE among hospitalized COVID-19 patients remains higher than those without COVID-19. In this study, pre-existing hypertension and cardiovascular diseases, but not high BMI or diabetes, were among the risk factors for VTE in this patient population. The study also raised the possibility that Hispanic COVID-19 patients are at higher risk of VTE. More proactive outpatient management of COVID-19 infection to prevent hospitalization can be a factor to reduce the incidence of VTE in these patients.
